# Atypical Proliferating Trichilemmal Cyst with Malignant Breast Skin Transformation: A Case Report and Review of the Literature

**DOI:** 10.1155/2016/7481569

**Published:** 2016-06-14

**Authors:** Marino Antonio Capurso-García, Verónica Bautista-Piña, Alan Pomerantz, Javier Andrés Galnares-Olalde, Ruben Blachman-Braun, Sergio Rodríguez-Rodríguez, Monica Goldberg-Murow

**Affiliations:** ^1^Departamento de Oncología Mamaria Quirúrgica, Instituto de Enfermedades de la Mama (IEM), La Fundación del Cáncer de Mama (FUCAM), 40980 Mexico City, Mexico; ^2^Facultad de Ciencias de la Salud, Universidad Anáhuac México Norte, 52786 Huixquilucan, MEX, Mexico; ^3^Departamento de Patología, Instituto de Enfermedades de la Mama (IEM), La Fundación del Cáncer de Mama (FUCAM), 40980 Mexico City, Mexico

## Abstract

Proliferating trichilemmal tumors (PTTs) are benign adnexal skin neoplasms that arise from the outer root sheath of the hair follicle. These tumors are most commonly observed on the scalp and occur, most of the time, in elderly women. Malignant transformation of these neoplasms is a rare event; less than 50 cases have been reported in the English medical literature. We present the case of a 39-year-old Hispanic woman with a tumor located on the skin of one of her breasts that in her third surgical procedure the histologic examination revealed the presence of a malignant proliferating trichilemmal tumor (MPTT). Furthermore, a review of the medical literature and a discussion of the clinical and pathologic features of this rare entity are provided.

## 1. Introduction

Proliferating trichilemmal tumors (PTTs) are benign adnexal skin neoplasms that arise from the outer root sheath of the hair follicle. Most of these tumors arise within the wall of a preexisting trichilemmal cyst [1]. Moreover, these tumors, from a histopathological standpoint, are very similar to a squamous cell carcinoma (SCC) [2]. PTTs were first described in 1966 by Wilson-Jones as epidermoid proliferating cysts [3]. However, it was not until 1995 that epidermoid proliferating cysts and PTTs were distinguished as different lesions [2].

PTTs encompass only 0.1% of all skin tumors. Additionally, most of the patients that present these lesions are elderly women, and in 90% of the cases these tumors occur on the scalp [4]. PTTs rarely exhibit malignant transformation (characterized by invading neighboring tissues and the presence of anaplasia and necrosis) into malignant proliferating trichilemmal tumors (MPTTs) [5–7], a fact substantiated by the presence of less than 50 cases of MPTTs reported in the English medical literature.

In this paper, the first case reported in Latin America regarding the malignant transformation of a PTT, we present the case of a woman with a tumor located on the skin of one of her breasts that in her third surgical procedure the histologic examination revealed the presence of a MPTT. In addition, a literature review and a discussion of the clinical and pathologic features of this rare entity are provided.

## 2. Case Report

A 39-year-old Hispanic woman was referred to our hospital due to the sudden appearance of a painful, mobile, fixed mass of about 5 cm in diameter in the internal upper quadrant of her right breast. The lesion was classified as a breast cyst, and a puncture of it was performed. Hyaline fluid was aspirated from the mass, with a subsequent decrease in its size. After two months, in the next follow-up, the growth reappeared. Therefore, the mass was surgically resected. The resected lesion was sent to the pathology department and a diagnosis of chronic granulomatous mastitis and fat necrosis was made.

**Figure 1:**
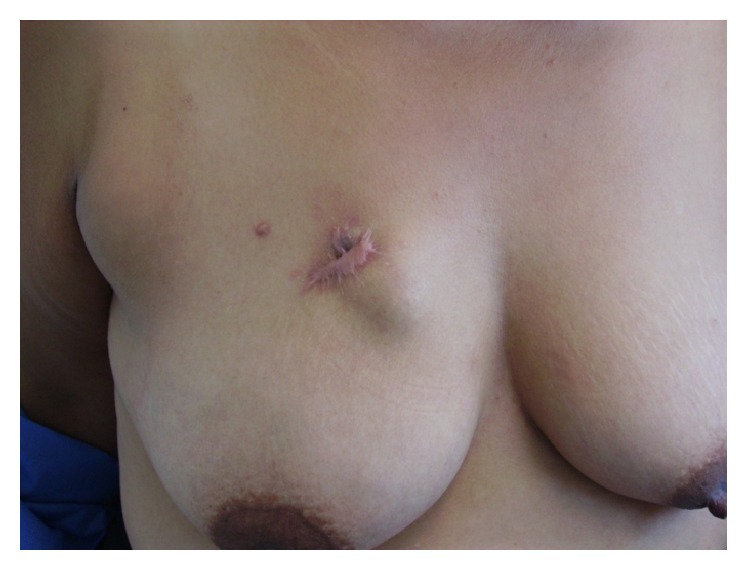
Photography of recurrent tumor in the right breast beneath the previous site of resection. Note the nodule under the scar deforming the overlying skin.

Three months after the surgery, the patient presented again for an evaluation due to the presence of a keloid scar of about 5 cm. When the breast was examined a 2 cm nodule located under the scar was palpated. The mass had well-defined borders and was not fixed to superficial or deep tissue (Figure 1). A breast ultrasound was performed, and it showed a cystic tumor of 2.33 cm, with well-defined borders and mixed echodensity. Due to the result of the ultrasound, the growth was described as a recurrent, complicated right breast skin cyst.

**Figure 2:**
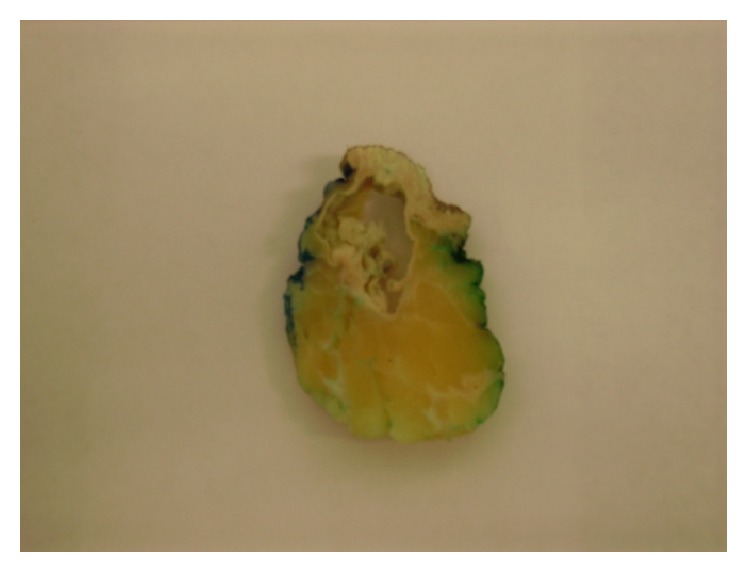
Pale yellow, firm, solid, lobulated, poorly defined mass, of 2.8 cm in length and 2.5 in width, covered by mature adipose tissue.

**Figure 3:**
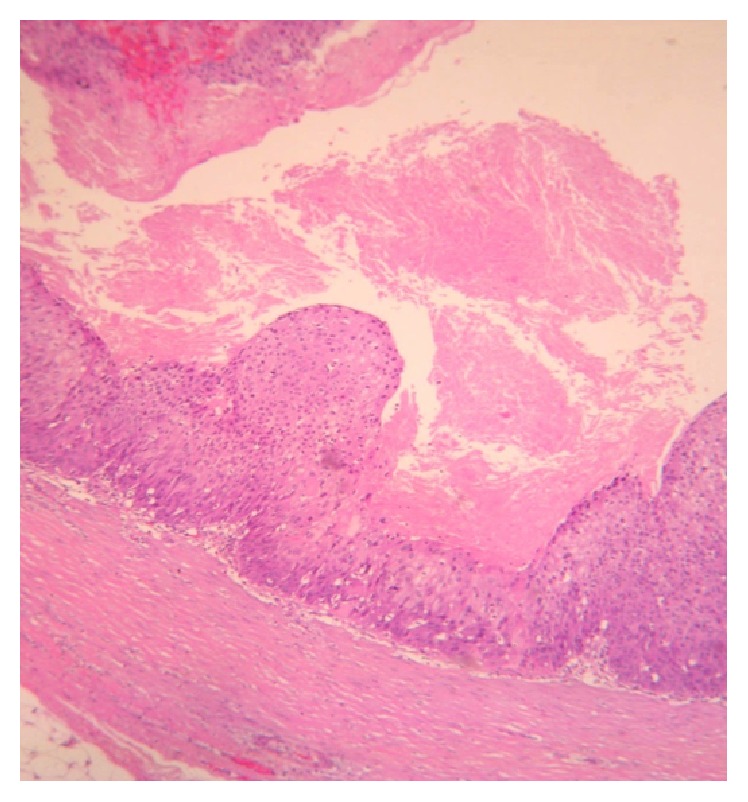
Cystic tumor portion where keratin amorphous deposits are observed (H and E, ×10).

**Figure 4:**
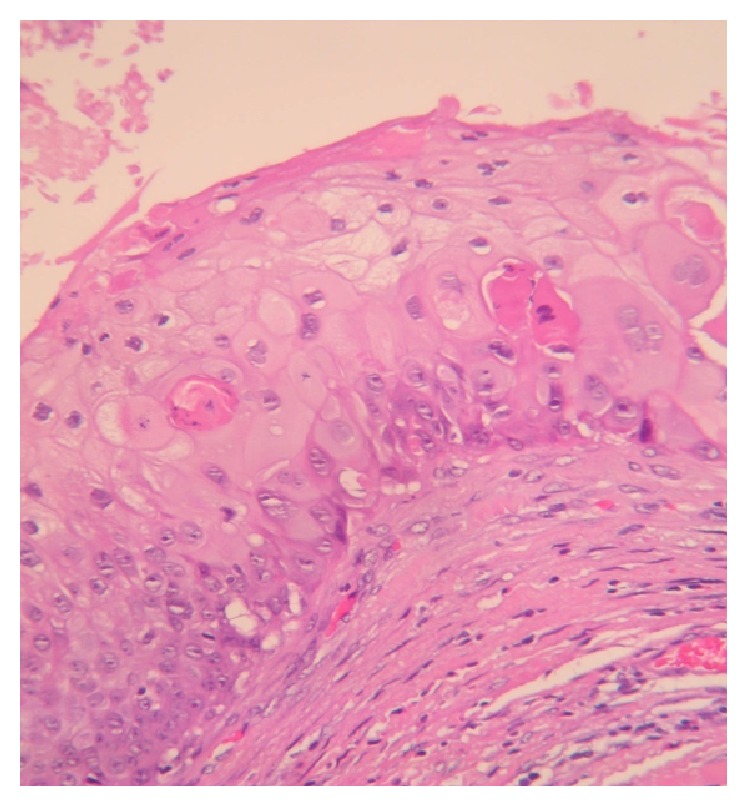
High magnification micrograph showing squamous epithelium keratinization (H and E, ×20).

**Figure 5:**
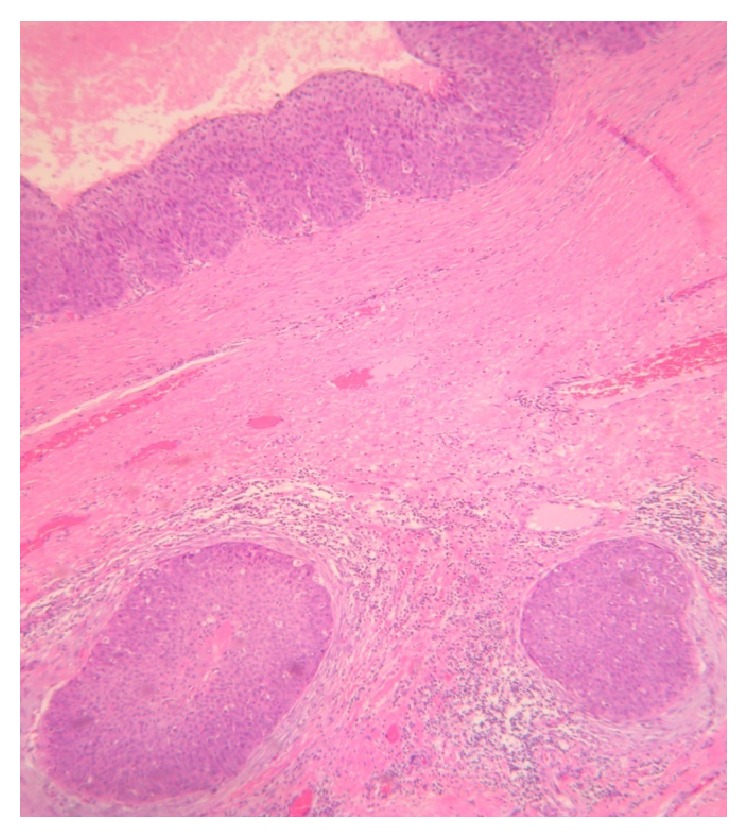
Photomicrograph showing epithelial cell nests in the stroma with desmoplastic reaction (H and E, ×10).

The patient underwent surgery in order to remove the tumorous growth. The lump was excised with amplified margins of 2 cm. The macroscopic appearance of the tumor was of a pale yellow, firm, solid, lobulated, poorly defined mass, of 2.8 cm in length and 2.5 in width, covered by mature adipose tissue (Figure 2). Microscopically, the tumor was described as a mixed tissue tumor, consisting of solid and cystic areas. The solid part of the neoplastic lesion was formed by squamous cells disposed as cordons, exhibiting peripheral palisades, with the presence of abrupt keratinization of the outer layers and focal calcifications. The cystic part presented a wall showing stratified squamous epithelium, multiple pleomorphic mitotic cells, and intraluminal keratin deposits (Figures 3 and 4). In some areas of the adjacent stroma, the pathologist observed squamous epithelial cell nests, with desmoplastic reaction and lymphocytic proliferation (Figure 5). The immunohistochemistry report was as follows: CD34 negative in the neoplastic cells, positive cytokeratin AE1/AE3, and positive p53 in 40% of the tumor. Finally, the diagnosis of MPTT was made, and the patient has been followed up since the diagnosis without any signs that the neoplasia has relapsed.

## 3. Discussion 

PTTs are rare, and the malignant transformation of these tumors is a rarer pathological finding. Moreover, only two cases in the literature have reported the presence of a MPTT over the skin of the breast [8, 9]. PTTs usually appear in women, 80–87% of the cases, between 27 and 83 years of age, with a peak in the sixth and seventh decades of life [4, 10]. They typically appear in sun-exposed areas and regions with abundant hair growth, thus explaining why these tumors appear most frequently on the scalp [5].

Histologically, PTTs are characterized by an abrupt transition of the nucleated epithelium to anucleated keratinized cells without a granular layer, a phenomenon known as trichilemmal keratinization. MPTTs can occur de novo but most often occur due to the malignant transformation of a PTT. A stepwise transformation of MPTTs has been described from an adenomatous to an epitheliomatous and then to a carcinomatous stage [11]. Additionally, in a study in which 76 patients were evaluated, the clinicopathological categorization of PTTs into three groups was proposed [4, 7].

*(i) Group 1 Tumors.* They are considered completely benign and present minimal nuclear atypia, trichilemmal keratinization, and stromal invasion with mononuclear cells, plasma cells, lymphocytes, and giant cells.

*(ii) Group 2 Tumors.* They are considered locally aggressive and present irregular and local invasive contours, with moderate cytological atypia, foci of single cell necrosis, and abrupt keratinization with desmoplastic stromal response.

*(iii) Group 3 Tumors.* They are considered malignant and present marked nuclear polymorphism, atypical mitosis, foci of single cell necrosis, abrupt keratinization, and lymphovascular invasion.

Even though the diagnosis of MPTTs is mostly done in a histopathological fashion, the use of immunohistochemistry can help to distinguish MPTTs from PTTs and SCCs. CD34 is a marker that is closely associated with trichilemmal keratinization, and because it is absent in SCCs and present in MPTTs it can help in differentiating one condition from the other (an important distinction because MPTTs have a greater chance of recurring and metastasizing) [7, 12]. As with this case, there have been reports of MPTTs where a negative staining with CD34 is observed. Moreover, some authors have postulated that a loss of CD34 staining is related to a decrease in the differentiation of the tumor. Ki-67 and p53 can help in the distinction of MPTTs from PTTs, as both markers are usually absent or dimly expressed in PTTs [7].

In order to evaluate possible local and distant metastases, imaging studies are warranted. It has been reported that on imaging studies MPTTs can manifest as either a cystic or a solid mass. Computed tomography scan is useful for the evaluation of focal bone involvement and erosion, and assessment of possible metastases; meanwhile, magnetic resonance imaging is reserved to evaluate soft tissue infiltration and signs of malignancy. Malignancy findings include poorly defined margins, penetration of tissue planes, and local invasion [1, 4].

As with other skin lesions, surgical excision remains the treatment of choice. Wide excision with margins of at least 1 cm is recommended [1, 4]. With a local recurrence rate of 3.7%, even for benign PTTs, Mohs micrographic surgery has been suggested as a better technique for the excision of MPTTs due to its superior margin control [13].

Due to the rarity of MPTTs, the efficacy of alternative treatments cannot be evaluated. In cases with distant metastases the use of CAV (cisplatin, adriamycin, and vindesine) chemotherapy, a regimen that has been used for advanced squamous cell carcinoma, has been attempted. Nonetheless, the results have not been promising [13]. Therefore, patients suspected of having this condition should be diagnosed and treated in an expedite manner, with a close follow-up after the excision of the tumor.

## 4. Conclusion

We report a case of a MPTT that occurred in an atypical location, which represented a clinical challenge in both the diagnosis and definitive treatment, due to the nonspecific clinical presentation and rarity of this condition. The recurrences that the patient presented may have occurred due to the fact that excision margins of less than 1 cm were used in the previous resection. Furthermore, it is important to state that the clinician should be alert to this diagnosis, especially in the presence of a cyst with recent rapid growth after remaining unchanged for a long time and tendency to recur after the tumor has been excised.
